# Age-related alterations in blood and colonic dendritic cell properties

**DOI:** 10.18632/oncotarget.7799

**Published:** 2016-03-01

**Authors:** Rakesh Vora, David Bernardo, Lydia Durant, Durga Reddi, Ailsa L. Hart, John M. E. Fell, Hafid O. Al-Hassi, Stella C. Knight

**Affiliations:** ^1^ Antigen Presentation Research Group, Imperial College London, Northwick Park and St. Mark's Campus, Harrow, UK; ^2^ London North West Healthcare NHS Trust, St. Mark's Campus, Harrow, UK; ^3^ Chelsea and Westminster Hospital NHS Foundation Trust, London, UK; ^4^ Gastroenterology Unit, Hospital Universitario de La Princesa and Instituto de Investigación Sanitaria Princesa (IIS-IP), Centro de Investigación Biomédica en Red de Enfermedades Hepáticas y Digestivas (CIBEREHD), Madrid, Spain

**Keywords:** mDC, pDC, children, aging, cytokines, BDCA-2, Gerotarget

## Abstract

**Background:**

Dendritic cells (DC) determine initiation, type and location of immune responses and, in adults, show decreased Toll-like receptors and some increased cytokine levels on ageing. Few studies in children have characterised DC or explored DC-related mechanisms producing age-related immune changes.

**Results:**

The pDC marker BDCA2 (but not CD123) was absent in pre-pubertal children and numbers of pDC decreased with age. Blood and colonic DC were more mature and activated in adults. Decrease in pDC numbers correlated with reduced GM-CSF levels with aging, but increasing IL-4 and IL-8 levels correlated with a more activated DC profile in blood. CXCL16 levels decreased with age.

**Methods:**

Blood and colonic DC phenotypes were determined in healthy adults and children by flow cytometry and correlated with aging. Blood DC were divided into plasmacytoid (pDC) and myeloid (mDC) while only mDC were identified in colon. Serum cytokine levels were determined by multiplex cytokine assays and correlated with DC properties.

**Conclusions:**

In children, lack of BDCA2, a receptor mediating antigen capture and inhibiting interferon induction, may be immunologically beneficial during immune development. Conversely, reduced pDC numbers, probably secondary to decreasing GM-CSF and increasing cytokine-induced maturation of DC are likely to determine deteriorating immunity with ageing.

## INTRODUCTION

Dendritic cells (DC) are central to the initiation of primary immune responses. They are the most potent antigen-presenting cells capable of stimulating naïve T cells and hence they are pivotal in the generation and control of adaptive immunity serving as a link between the innate and adaptive immune system [1]. DC can be subdivided into myeloid (mDC) and plasmacytoid (pDC). mDC capture antigens in the periphery and then migrate to the lymphoid organs to initiate immunity, whereas pDC are found in the thymic medulla and lymph node T cell areas and contribute to anti-viral responses [2].

Few studies have characterised DC in children usually with contradictory results [3-6]. However, studies examining different DC subsets showed that numbers of circulating pDC (but not mDC) are inversely correlated with age [6-8] while the phenotype and antigen presenting capacity of circulating and monocyte-derived DC is not affected with age [9]. Nevertheless, a recent study has described specific changes in the activation of the AKT kinase pathway resulted in an impaired migration capacity, decreased ability to uptake and process antigens and increased cytokine production by mDC in aged subjects [10-12]. Therefore, given the lack of studies addressing age-related effect on DC properties, here we have characterized blood and colonic DC in healthy children and adults to investigate age-related changes.

## RESULTS

### Characterization of human blood and colonic DC

**Figure 1:**
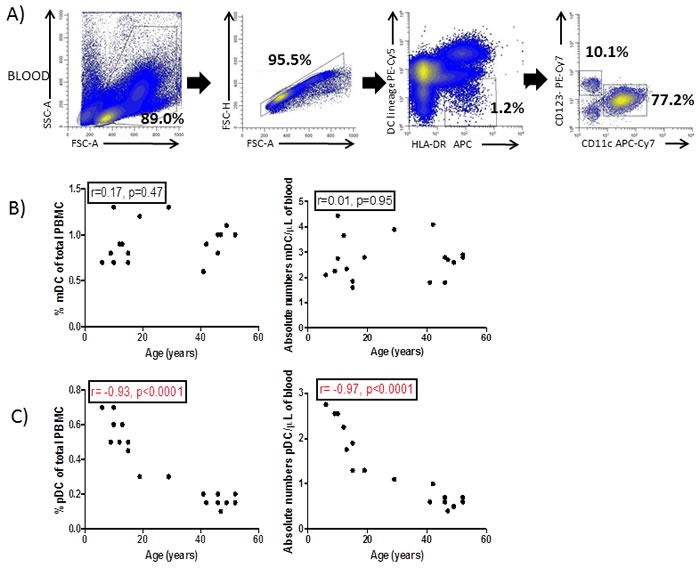
Blood and colonic DC identification **A.** Forward and side scatter plot of peripheral blood mononuclear cells (PBMC) and subsequent doublet discrimination. Blood dendritic cells (DC) were identified as HLA-DR^+^lineage^−^ (CD3,CD14,CD16,CD19,CD34) and further divided into myeloid (CD11c^+^,mDC) and plasmacytoid (CD123^+^,pDC). **B.** Percentage and absolute numbers of mDC and **C.** pDC were correlated with the age of the donors. **D**. Colonic DC were identified with singlet viable lamina propria mononuclear cells (LPMC) based on the forward and side scatter properties of the cells and identified within CD45^+^ cells as HLA-DR^+^lineage^−^CD11c^+^. **E**. Percentage of colonic DC (referred to total LPMC) were also correlated with the age of the donor. Pearson's correlation analysis and *p*-value is displayed. *P*<0.05 was considered as statistically significant.

Total DC were identified within single viable PBMC (based on the forward/side scatter properties of the cells) that were HLA-DR^+^lineage (CD3, CD14, CD16, CD19, CD34)^−^ as previously described [13, 14]. DC were subsequently divided into mDC (CD11c^+^CD123^−^) and pDC (CD11c^−^CD123^+^) (Figure 1A).

The proportion (relative to total PBMC) and absolute numbers of mDC were not affected by the age of the donor (Figure 1B). However, both proportion (*r* = 0.93, *p* < 0.0001) and absolute numbers (*r* = 0.97, *p* < 0.0001) of pDC decreased with age (Figure 1C).

Colonic DC were identified within single viable LPMC (based on the forward/side scatter properties of the cells) as leukocytes (CD45^+^) which were HLA-DR^+^lineage^−^. Only CD11c^+^ mDC were identified within colonic DC (Figure 1D) and their frequency was not affected by the age of the donor (Figure 1E).

### BDCA2 (CD303) is absent on blood pDC in pre pubertal children

**Figure 2:**
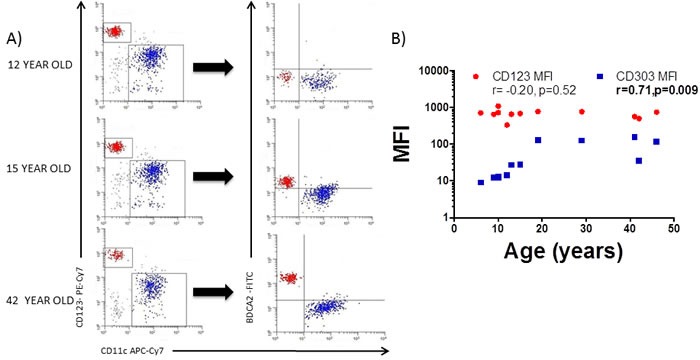
BDCA2 is absent on pediatric blood pDC **A.** Blood myeloid (mDC) and plasmacytoid (pDC) dendritic cells were identified as in Figure 1A and backgated for the analysis of BDCA2 (CD303) in a 12 year old, a 15 year old and a 42 year old adult healthy control. Pooled data is shown in **B.** Pearson's correlation analysis and *p*-value is displayed. *P* < 0.05 was considered as statistically significant.

Having confirmed that blood pDC levels were affected by age (Figure 1C), we next determined if expression of the markers typically used to identify them (i.e. CD123 and BDCA2) were also affected by the age of the donor. The expression of CD123 was unaffected by the age (Figure 2A) when determined as the mean fluorescence index (MFI, *r* = −0.20, *p* = 0.52) (Figure 2B). However, BDCA2 expression was absent on CD11c^−^CD123^+^ pDC from pre pubertal pediatric patients (Figure 2A) with its expression displaying a positive correlation (*r* = 0.71, *p* = 0.009) with the age of the donor (Figure 2B).

### Blood DC immunophenotyping

**Figure 3:**
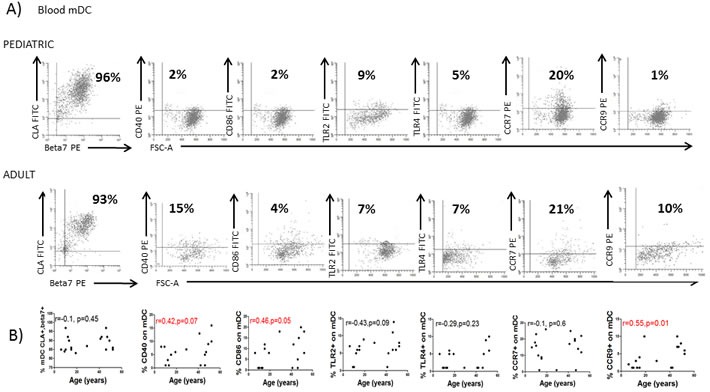
Blood DC changes with the age **A.** Blood myeloid dendritic cells (mDC) were identified as in Figure 1A and characterized for the expression of several markers in a representative healthy children and a healthy adult. Pooled data and correlation with the age of the donor is shown in **B. C.** Blood plasmacytoid dendritic cells (pDC) were also identified as in Figure 1A and characterized for the expression of several markers in a representative healthy children and a healthy adult. Pooled data and correlation with the age of the donor is shown in **D.** In all cases, the expression of each individual marker is shown as percentage (%). Pearson's correlation analysis and *p*-value is displayed. *P* < 0.05 was considered as statistically significant.

Since both circulating pDC numbers and their BDCA2 expression are influenced by the age of the donor, we next characterised DC phenotype in both healthy adults and children to identify other potential markers which may be age-influenced. Blood mDC and pDC were thus characterized for the expression of the mucosal (Integrin beta-7; β7), skin (Cutaneous lymphocyte antigen; CLA), lymph-node (C-C chemokine receptor; CCR7) and small bowel (C-C chemokine receptor 9; CCR9) homing markers, together with their activation status (CD40 and CD86) and expression of pattern recognition receptors (Toll-like receptors (TLR)2 and 4) (Figure 3). Blood mDC (Figure 3A and 3B) and pDC (Figure 3C and 3D) from the adult population were more mature (higher CD40 and CD86 expression) with higher small bowel homing capacity (CCR9). Additionally, adult pDC had lower lymph-node homing capacity (lower expression of CCR7) and higher potential capacity to recognize microbial antigens (higher levels of TLR2) (Figure 3C and 3D).

### Colonic DC characterization

**Figure 4:**
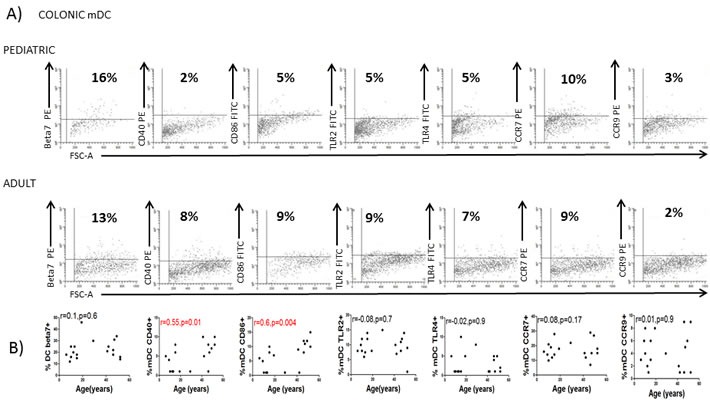
Colonic DC changes with the age **A.** Colonic dendritic cells (DC) were identified as in Figure 1D and characterized for the expression of several markers in a representative healthy children and a healthy adult. Pooled data and correlation with the age of the donor is shown in **B.** Pearson's correlation analysis and *p*-value is displayed. *P* < 0.05 was considered as statistically significant.

We next studied whether the age-related properties of circulating DC were also reflected in other tissues so human colonic DC were also characterized. In agreement with observations of blood mDC and pDC, colonic DC from the same donors were more mature (CD40 and CD86) in the adult population (Figure 4A and 4B) confirming a lower activation status of circulating and colonic DC in the pediatric population.

### Blood cytokines and chemokines

Next, we studied the potential mechanisms that may account for changes in DC phenotype with aging by determining serum concentration of several cytokines and chemokines. Our results revealed that the adult population had higher serum levels of IL-4 and IL-8 coupled with lower levels of CXCL16 and GM-CSF while 36 other soluble mediators remained unchanged with age (Table 1).

**Table 1:** Cytokine levels in the plasma

Cytokines	Children Cytokine concentration pg/ml		Adults Cytokine concentration pg/ml		*r*-value	*p*-value
	Mean	Standard deviation	Mean	Standard deviation		
CCL1	12.95	1.44	13.16	0.63	0.18	0.62
CCL11	12.36	4.18	14.28	3.01	0.23	0.49
CCL15	1194	386.8	2615	1756	0.64	0.12
CCL17	13.45	6.21	12.69	13.34	−0.15	0.76
CCL19	10.44	4.66	11.36	9.60	0.08	0.84
CCL20	2.56	0.43	3.07	1.50	0.31	0.37
CCL21	614.1	259.9	531.4	290.2	−0.17	0.62
CCL24	4.89	2.33	9.63	6.48	0.48	0.18
CCL25	62	13.99	64.10	18.90	0.14	0.71
CCL26	20.21	11.79	14.31	6.62	−0.34	0.36
CCL27	51.22	33.74	36.75	20.66	−0.33	0.38
CX3CL1	23.13	10.58	18.91	7.01	−0.27	0.40
CXCL1	32.24	13.03	32.66	7.27	0.05	0.90
CXCL12	76.88	29.16	152.2	69.46	0.56	0.11
CXCL13	2.19	0.43	1.91	1.20	−0.12	0.75
CXCL16	161.2	51.70	105.2	22.30	***-0.62***	***0.05***
CXCL2	13.83	4.10	18.06	3.26	0.52	0.17
CXCL5	96.4	31.09	55.98	9.68	−0.72	0.10
CXCL6	5.13	1.71	4.13	0.56	−0.35	0.48
GM-CSF	31.65	8.46	9.08	11.95	***-0.74***	***0.01***
IFN gamma	***		***			
IL-10	2.77	1.31	2.62	0.62	−0.01	0.96
IL-16	194.8	66.78	174.1	104.8	−0.04	0.91
IL-1Beta	0.11	0.08	0.13	0.06	0.05	0.91
IL-2	5.81	1.44	4.76	0.72	−0.39	0.25
IL-4	1.10	0.53	2.91	1.28	***0.79***	***0.005***
IL-6	4.13	3.79	1.08	0.43	−0.56	0.09
IL-8	1.39	1.05	9.92	7.93	***0.69***	***0.01***
IP10	5.18	4.26	11.1	6.54	0.48	0.18
ITAC	9.80	5.32	8.79	6.57	−0.16	0.66
MCP1	8.97	7.07	4.85	1.97	−0.32	0.33
MCP2	3.93	3.26	7.18	4.03	0.46	0.17
MCP3	9.46	1.97	14.77	5.13	0.54	0.20
MCP4	9.45	3.19	18.34	12.25	0.41	0.30
MDC	93.88	25.67	108.5	35.19	0.25	0.47
MIF	116.7	87.68	46.11	42.62	−0.42	0.22
MIG	32.26	10.75	22.23	15.35	−0.42	0.24
MIP1alpha	0.95	0.61	0.61	0.28	−0.31	0.44
MPIF1	28.84	10.37	49.82	30.03	0.54	0.10
TNF Alpha	3.44	1.39	3.39	1.17	−0.02	0.94

Statistically significant differences for each cytokine/chemokine after Pearson's correlation analysis with the age are shown in bold italics

*** = below detection limit, pg/ml=picograms/millilitre

Finally, we determined whether such differences in cytokine/chemokine levels correlated with age-related changes in DC phenotype (Table 2). The lower levels of pDC with aging correlated with GM-CSF concentration and inversely correlated with IL-4 and IL-8 concentrations. BDCA2 MFI on pDC, on the contrary, displayed a positive correlation withIL-4 and IL-8 levels. CD40 on pDC and TLR2 expression on mDC correlated with IL-4 and IL-8 concentrations while CD86 correlated with IL-8 concentrations and inversely with GM-CSF levels. Changes in CCR7 expression on mDC correlated with IL-4 and IL-8 levels and inversely with GM-CSF and CXCL16 levels. CCR7 expression on pDC correlated with GM-CSF levels. CCR9 expression correlated with IL-4 and IL-8 levels. β7 and CLA did not correlate with any of the cytokine or chemokine levels. As for colonic DC, CD40 expression correlated inversely with GM-CSF. The rest of the DC phenotypic markers did not correlate with any cytokine or chemokine levels. Together the evidence suggested that the circulating levels of IL-4, IL-8 and GM-CSF are the main soluble factors influencing the age-related properties of blood and colonic DC (Table 2).

**Table 2:** Blood cytokines and chemokines correlation with DC markers

DC numbers &markers	Cytokines							
	IL-4		IL-8		GM-CSF		CXCL16	
	*r*-value	*p*-value	*r*-value	*p*-value	*r*-value	*p*-value	*r*-value	*p*-value
Blood mDC%	0.03	0.92	0.24	0.26	−0.35	0.31	−0.5	0.1
Blood pDC %	***−0.7***	***0.02***	***−0.69***	***0.01***	***0.80***	***0.004***	***0.41***	***0.23***
Colon mDC%	−0.14	0.68	−0.47	0.13	0.32	0.36	0.21	0.54
Blood mDC absolute numbers	−0.3	0.29	−0.1	0.69	−0.08	0.81	0.05	0.87
Blood pDC absolute numbers	***−0.83***	***0.002***	***−0.71***	***0.01***	***0.65***	***0.03***	***0.03***	***0.94***
CD123 MFI pDC	0.11	0.8	−0.07	0.87	−0.04	0.9	−0.5	0.3
BDCA2 MFI pDC	***0.96***	***0.0005***	***0.87***	***0.0009***	−0.7	0.12	−0.21	0.68
Beta7 blood mDC	0.61	0.07	0.36	0.27	−0.07	0.83	−0.52	0.11
Beta7 blood pDC	0.05	0.87	0.6	0.07	−0.2	0.53	0.4	0.24
Beta7 colon mDC	0.06	0.9	−0.73	0.5	−0.67	0.14	0.2	0.73
CLA blood mDC	0.36	0.3	0.07	0.82	0.5	0.13	−0.3	0.39
CLA blood pDC	−0.13	0.7	−0.11	0.72	0.2	0.57	−0.02	0.95
CLA colon mDC	0.46	0.42	0.59	0.21	−0.71	0.11	0.36	0.54
CD40 blood mDC	0.48	0.15	0.5	0.11	−0.16	0.64	−0.09	0.78
CD40 blood pDC	***0.73***	***0.01***	***0.98***	***0.0001***	−0.05	0.13	−0.37	0.28
CD40 colon mDC	−0.65	0.22	−0.38	0.44	−0.79	0.05	−0.13	0.83
CD86 blood mDC	0.26	0.45	0.55	0.07	−0.45	0.18	−0.19	0.59
CD86 blood pDC	0.41	0.23	***0.69***	***0.01***	***−0.69***	***0.02***	−0.45	0.18
CD86 colon mDC	−0.72	0.16	−0.35	0.49	−0.71	0.09	−0.13	0.83
TLR2 blood mDC	***0.72***	***0.01***	***0.79***	***0.003***	−0.32	0.36	−0.15	0.66
TLR2 blood pDC	***0.85***	***0.001***	***0.81***	***0.002***	−0.37	0.28	−0.45	0.18
TLR2 colon mDC	−0.48	0.40	−0.45	0.36	−0.49	0.31	−0.08	0.89
TLR4 blood mDC	−0.12	0.72	−0.15	0.64	0.33	0.34	0.59	0.07
TLR4 blood pDC	0.19	0.58	0.38	0.23	−0.22	0.53	0.0008	0.99
TLR4 colon mDC	−0.28	0.64	−0.39	0.43	−0.51	0.29	−0.13	0.83
CCR7 blood mDC	***0.65***	***0.04***	***0.69***	***0.01***	***−0.84***	***0.001***	***−0.63***	***0.04***
CCR7 blood pDC	−0.25	0.47	−0.30	0.35	***0.68***	***0.02***	0.57	0.08
CCR7 colon mDC	0.28	0.63	0.07	0.89	−0.56	0.23	0.21	0.72
CCR9 blood mDC	***0.85***	***0.001***	***0.57***	***0.06***	−0.40	0.24	−0.35	0.31
CCR9 blood pDC	***0.82***	***0.003***	***0.83***	***0.001***	−0.36	0.29	−0.55	0.09
CCR9 colon mDC	−0.05	0.92	0.20	0.69	−0.32	0.53	−0.38	0.51

Statistically significant differences for each DC parameter after Pearson's correlation analysis with IL-4, IL-8, GM-CSF and CXCL16 concentration are shown in bold italics.

## DISCUSSION

Here we show that expression of BDCA2 (CD303) only appears on blood pDC at around the age of puberty. In addition, a decrease in blood pDC numbers with age coupled with general increases in maturation status of both colonic and blood pDC and mDC is also described. The findings suggest that the DC populations provide evidence both of maturation of the immune system in young children as well as supporting the view that aging in older people is a process of general deterioration of immunity; development of an on-going low-grade inflammation may be an underlying cause of mortality and age-related diseases [15].

BDCA2 is commonly used as a pDC marker so that its absence in pre-pubertal children might lead to practical problems of pDC identification in the pediatric populations when using this marker instead of CD123. However, functional changes related to lack of BDCA2 may be more significant; BDCA2 signalling leads to reduced levels of transcripts for type-1 interferon genes [16] suggesting that production of such cytokines could be increased in pDC during childhood. Therefore, the lack of BDCA2 on pDC during childhood could have beneficial effects given that children have high propensity for viral infections so need this enhanced ability to make innate IFN due to lack of T cell memory. However, these potential functional differences between adult and pediatric pDC warrant further study which are limited by ethical considerations when studying healthy children. The mechanisms responsible for aging-related chronic inflammation are also not well understood. Here, we provide some bases for deteriorating immunity by describing a specific reduction of circulating pDC with age coupled with an increased maturation status of both blood and colonic DC.

Blood pDC numbers decreased with age suggesting a partial dysregulation of circulating DC. There was also an increased expression of TLR2 on pDC and increased expression of CD40 and CD86 in both mDC and pDC suggesting an increase in the general activation profile of DC with aging. Reports on young and aged adults have not shown similar increases in activation profile [17]. However, there was no comparison of DC in children and adults which was the focus of our study. There is reduced expression of TLR1,3,7,8 in aged individuals when compared with young adults [18, 19] and increased production of pro-inflammatory cytokines (IL-6 and IL-18) and lower amounts of anti-inflammatory mediators including IL-10 with aging in adults [17, 20, 21]. The studies in the literature thus support our findings that aging does result in a more pro-inflammatory DC profile. In addition,, the increased maturation status of DC was not restricted to blood pDC but was also found on blood mDC and colonic DC which suggests a more systemic activation in both blood and tissues.

In order to gain insights into possible mechanism at play in the development of DC changes with age we studied serum levels of 40 cytokines/chemokines. IL-4 and IL-8 displayed a positive correlation with aging as opposed to CXCL16 and GM-CSF which displayed a negative correlation. The increase of IL-4 and IL-8 with age is in keeping with previously published literature [17, 22] suggesting that the general body milieu becomes more pro-inflammatory with age. Indeed, IL-4 and IL-8 were the cytokines which displayed the biggest correlation with the age of the donor (Table 1) and correlated with most of the age-induced changes of DC properties (Table 2) including the lower numbers of circulating pDC with aging and the lack of BDCA2 expression of pre-pubertal circulating pDC suggesting they are the main cytokines influencing the age-related changes displayed by DC. However, adult mDC and pDC from healthy adult volunteers (mean age 38.7±4.2 years) did not alter the expression of any of the studied markers following short term *in vitro* stimulation with IL-4, IL-8 or GM-CSF (data not shown) suggesting that such changes may be initiated at a younger stage or require more persistent exposure.

In contrast to IL-4 and IL-8, which displayed a positive correlation with age, GM-CSF showed a negative correlation with age. GM-CSF is used to promote monocyte differentiation *in vitro* into monocyte-derived DC (MoDC) [23-26]. Given that lower pDC numbers with aging also correlated with lower levels of GM-CSF, GM-CSF might affect circulating pDC levels. Finally, CXCL16 was significantly higher in children. CXCL16 is an age and organ-dependent chemokine regulated by microbial exposure during early life [27]; lower levels of bacterial exposure during early life correlate with higher levels of CXCL16 [28, 29]. The high CXCL16 in children might be in keeping with increased antibiotic exposure of children in the current era. However the age-related changes on CXCL16 were borderline (Table 1) so we cannot conclude that there is a direct influence.

In summary, we have described age-related changes to blood and colonic DC numbers and phenotype, particularly acquisition of the marker BDCA-2 and reduced numbers of circulating pDC with age. It will be important to determine how these changes in DC phenotype affect their function during our lifetime. The results showing lower BDCA2 on pDC in children could also be a basis for age-related functional differences in responsiveness to interferons in children.

## MATERIALS AND METHODS

### Blood and colonic tissue samples

Blood samples and distal colonic biopsies were obtained from 8 children (age range 6-15 years, 6 males) while undergoing colonoscopy for painless rectal bleeding for suspected benign polyp. The endoscopies were macroscopically and histologically normal apart from 3 patients showing isolated rectal polyps. All the children were recruited over a 2 year period in a pediatric gastroenterology department at Chelsea & Westminster Hospital (London, UK).

Blood and distal colonic biopsies were also obtained form 10 adults (Age range 19- 52 years, 4 males) when undergoing endoscopy for change in bowel habit from an adult gastroenterology centre at St Mark's Hospital (London, UK). The endoscopies were macroscopically and histologically normal in all the 10 patients.

Informed consent was obtained from all patients/parents/legal guardians and the protocol was approved by the National Research ethics committee and the local research and development committee. The exclusion criteria were: diagnosed or suspected chromosomal abnormality, major medical illness or immunocompromised states.

### Blood dendritic cells

Serum samples were immediately frozen at −80°C following blood extraction. Peripheral blood mononuclear cells (PBMC) were subsequently obtained by centrifugation of 10 ml of human peripheral blood over Ficoll Paque Plus (GE Healthcare, UK) at 800g, for 30 minutes at room temperature and harvesting the buffy coat layer from the interface. The PBMC were washed twice (centrifugation 650g, 10 minutes and 450g, 5 minutes) in RPMI 1640 Dutch modification (Sigma-Aldrich, Dorset, England).

### Colonic biopsies

Five colonic mucosal biopsy specimens from the distal colon were taken per patient. Biopsy specimens were collected in ice-chilled complete medium (RPMI 1640 Dutch modification supplemented with 10% fetal calf serum, 2mM L-glutamine, 25 μg/mL gentamicin and 100 U/mL penicillin/streptomycin) and processed within an hour. Mucus and faeces were removed from the biopsies using 1 mmol/L dithiothreitol (Sigma-Aldrich) in Hank's balanced salt solution (Gibco BRL, Paisley, Scotland) for 20 minutes in T25 flasks. The epithelium was removed using two 30-minute treatments with 1 mmol/L EDTA in calcium- and magnesium-free Hank's balanced salt solution at 37°C with gentle agitation. The biopsy specimens were cultured (1 biopsy per well in 1ml of complete medium) in a CO_2_ incubator (BioHit- 37°c, 5% CO_2_, high humidity) for 24 hours as previously described (14). Lamina propria mononuclear cells (LPMC) released from the tissue samples were passed through a 100μm cell strainer and washed twice with complete medium (Centrifugation at 600g, 5 minutes).

### Antibody labelling and flow cytometry

A minimum of 100,000 PBMC or LPMC were washed in fluorescence activated cell sorter (FACS) buffer (phosphate buffered saline (PBS) with 1 mmol/L EDTA and 0.02% sodium azide) and labelled on ice for 20 minutes with monoclonal antibodies at predetermined optimal concentrations. Details of the specific antibodies (including clone and conjugated fluorochromes) can be found in Supplementary Table 1. Cells were then washed twice (450g, 5minutes) in FACS buffer and subsequently fixed with 1% paraformaldehyde (1% PFA). Cells were acquired within 48 hours on a FACS Canto-II flow cytometer (Becton-Dickinson, UK). Data analysis was carried out using Winlist™ software (Verity Software House, Maine, USA). The proportion of cells expressing any given surface marker of interest was determined by comparing fluorescence to that of an isotype-matched control antibody. Flow-count fluorospheres™ (Beckman Coulter, UK) were also added to all samples to determine absolute numbers.

### Cytokine analysis

Cytokine and chemokine levels of 40 different metabolites were determined in the serum samples using the multiplex immunoassay Bio-Plex Pro TM Human chemokine panel (BIO-RAD) on a Luminex MAGPIX system (Merck Millipore). Determined cytokines and their detection limits (following manufacturer's instructions) are detailed in Supplementary Table 2.

### Statistical analysis

Statistical analyses were carried out using GraphPad software (La Jolla-CA, USA). Pooled data are expressed as mean values +/− SEM. Non parametric tests (Mann Whitney Rank-sum tests and Pearson's correlation analysis) were used and *p* < 0.05 was considered significant.

## SUPPLEMENTARY MATERIAL TABLES



## References

[1] Steinman RM (2006). Linking innate to adaptive immunity through dendritic cells. Novartis Foundation symposium.

[2] Ezzelarab M, Thomson AW (2011). Tolerogenic dendritic cells and their role in transplantation. Seminars in immunology.

[3] Jyonouchi H, Cui C, Geng L, Yin Z, Fitzgerald-Bocarsly P (2010). Age-dependent changes in peripheral blood dendritic cell subsets in normal children and children with specific polysaccharide antibody deficiency (SPAD). European journal of pediatrics.

[4] Teig N, Moses D, Gieseler S, Schauer U (2002). Age-related changes in human blood dendritic cell subpopulations. Scandinavian journal of immunology.

[5] Hagendorens MM, Ebo DG, Schuerwegh AJ, Huybrechs A, Van Bever HP, Bridts CH (2003). Differences in circulating dendritic cell subtypes in cord blood and peripheral blood of healthy and allergic children. Clinical and experimental allergy: journal of the British Society for Allergy and Clinical Immunology.

[6] Vakkila J, Thomson AW, Vettenranta K, Sariola H, Saarinen-Pihkala UM (2004). Dendritic cell subsets in childhood and in children with cancer: relation to age and disease prognosis. Clinical and experimental immunology.

[7] Shodell M, Siegal FP (2002). Circulating, interferon-producing plasmacytoid dendritic cells decline during human ageing. Scandinavian journal of immunology.

[8] Perez-Cabezas B, Naranjo-Gomez M, Fernandez MA, Grifols JR, Pujol-Borrell R, Borras FE (2007). Reduced numbers of plasmacytoid dendritic cells in aged blood donors. Experimental gerontology.

[9] Steger MM, Maczek C, Grubeck-Loebenstein B (1996). Morphologically and functionally intact dendritic cells can be derived from the peripheral blood of aged individuals. Clinical and experimental immunology.

[10] Agrawal A, Gupta S (2011). Impact of aging on dendritic cell functions in humans. Ageing research reviews.

[11] You J, Dong H, Mann ER, Knight SC, Yaqoob P (2014). Probiotic modulation of dendritic cell function is influenced by ageing. Immunobiology.

[12] You J, Dong H, Mann ER, Knight SC, Yaqoob P (2013). Ageing impairs the T cell response to dendritic cells. Immunobiology.

[13] Al-Hassi HO, Bernardo D, Murugananthan AU, Mann ER, English NR, Jones A (2013). A mechanistic role for leptin in human dendritic cell migration: differences between ileum and colon in health and Crohn's disease. Mucosal immunology.

[14] Mann ER, Bernardo D, English NR, Landy J, Al-Hassi HO, Peake ST (2015). Compartment-specific immunity in the human gut: properties and functions of dendritic cells in the colon versus the ileum. Gut.

[15] Weiskopf D, Weinberger B, Grubeck-Loebenstein B (2009). The aging of the immune system. Transplant international.

[16] Dzionek A, Sohma Y, Nagafune J, Cella M, Colonna M, Facchetti F (2001). BDCA-2, a novel plasmacytoid dendritic cell-specific type II C-type lectin, mediates antigen capture and is a potent inhibitor of interferon alpha/beta induction. The Journal of experimental medicine.

[17] Ciaramella A, Bizzoni F, Salani F, Vanni D, Spalletta G, Sanarico N (2010). Increased pro-inflammatory response by dendritic cells from patients with Alzheimer's disease. Journal of Alzheimer's disease: JAD.

[18] Panda A, Qian F, Mohanty S, van Duin D, Newman FK, Zhang L (2010). Age-associated decrease in TLR function in primary human dendritic cells predicts influenza vaccine response. Journal of immunology.

[19] Jing Y, Shaheen E, Drake RR, Chen N, Gravenstein S, Deng Y (2009). Aging is associated with a numerical and functional decline in plasmacytoid dendritic cells, whereas myeloid dendritic cells are relatively unaltered in human peripheral blood. Human immunology.

[20] Vasto S, Candore G, Balistreri CR, Caruso M, Colonna-Romano G, Grimaldi MP (2007). Inflammatory networks in ageing, age-related diseases and longevity. Mechanisms of ageing and development.

[21] Gangemi S, Basile G, Merendino RA, Minciullo PL, Novick D, Rubinstein M (2003). Increased circulating Interleukin-18 levels in centenarians with no signs of vascular disease: another paradox of longevity?. Experimental gerontology.

[22] Man AL, Bertelli E, Rentini S, Regoli M, Briars G, Marini M, Watson AJ (2015). Age-associated modifications of intestinal permeability and innate immunity in human small intestine. Clin Sci (Lond).

[23] Daro E, Pulendran B, Brasel K, Teepe M, Pettit D, Lynch DH (2000). Polyethylene glycol-modified GM-CSF expands CD11b(high)CD11c(high) but notCD11b(low)CD11c(high) murine dendritic cells *in vivo*: a comparative analysis with Flt3 ligand. Journal of immunology.

[24] Vremec D, Shortman K (1997). Dendritic cell subtypes in mouse lymphoid organs: cross-correlation of surface markers, changes with incubation, and differences among thymus, spleen, and lymph nodes. Journal of immunology.

[25] van de Laar L, Coffer PJ, Woltman AM (2012). Regulation of dendritic cell development by GM-CSF: molecular control and implications for immune homeostasis and therapy. Blood.

[26] Louis C, Cook AD, Lacey D, Fleetwood AJ, Vlahos R, Anderson GP (2015). Specific Contributions of CSF-1 and GM-CSF to the Dynamics of the Mononuclear Phagocyte System. Journal of immunology.

[27] Olszak T, An D, Zeissig S, Vera MP, Richter J, Franke A (2012). Microbial exposure during early life has persistent effects on natural killer T cell function. Science.

[28] Neish AS, Denning TL (2010). Advances in understanding the interaction between the gut microbiota and adaptive mucosal immune responses. F1000 biology reports.

[29] Shaw SY, Blanchard JF, Bernstein CN (2010). Association between the use of antibiotics in the first year of life and pediatric inflammatory bowel disease. The American journal of gastroenterology.

