# Supplementary material for: Age-related alterations in blood and colonic dendritic cell properties
Source: Oncotarget. 2016 Mar 1;7(11):11913–22. doi: 10.18632/oncotarget.7799 (PMC4914258; doi:10.18632/oncotarget.7799)
Supplement: Supplementary file 1 [file oncotarget-07-11913-s001.pdf]

Age-related alterations in blood and colonic dendritic cell properties

Supplementary Material

Supplementary Table 1: Antibodies and flow cytometry

Antibody Specificity	Clone	Conjugate	Manufacturer
β7	FIB504	PE	BD
CCR7	150503	PE	R&D
CCR9	248621	PE	R&D
CD3	UCHT1	PE-Cy5	BD
CD11c	3.9	PE-Cy7	eBioscience
CD14	61D3	PE-Cy5	Serotec
CD16	3G8	PE-Cy5	BD
CD19	HIB19	PE-Cy5	BD
CD34	581	PE-Cy5	BD
CD40	LOB7/6	PE	Serotec
CD86	BU63	FITC	Serotec
CD123	6H6	PE-Cy7	eBioscience
CD303 (BDCA2)	AC144	FITC	Miltenyi
CLA	HECA-452	FITC	BD
HLA-DR	G46-6	APC	BD
CD11C	Bu15	APC-Cy7	Biolegend
TLR-2	TLR2.3	FITC	Serotec
TLR-4	HTA125	FITC	Serotec

Supplementary Table 2: Bio-Plex Pro™ Human Chemokine Panel, 40-Plex #171-AK99MR2

Cytokines	Detection limits (lower and upper limit) in picograms/ml
CCL1	12.46 – 951.09
CCL11	1.14 – 981.32
CCL15	4.79 – 4669.23
CCL17	1.14 – 981.32
CCL19	2.99 – 2776.12
CCL20	1.64 – 387.87
CCL21	9.87 – 610.77
CCL24	21.23 – 1245.46
CCL25	104.87 – 39686.33
CCL26	2.43 – 1956.08
CCL27	1.31 – 5385.13
CX3CL1	3.25 – 3344.75
CXCL1	13.64 – 2927.80
CXCL12	9.56 – 49441.41
CXCL13	0.51 – 408.27
CXCL16	2.20 – 456.51
CXCL2	5.91 – 1358.78
CXCL5	159.58 – 168556.11
CXCL6	3.11 – 3352.53
GM-CSF	7.67 – 32449.44
IFN gamma	111.73 – 9315.84
IL10	1.19 – 1146.35
IL16	8.07 – 32619.51
IL1Beta	2.21 – 2347.78
IL2	3.19 – 11337.47
IL4	1.17 – 1068.03
IL6	9.52 – 662.36
IL8	0.47 – 477.12
IP10	1.13 – 1169.36
ITAC	0.20 – 184.40
MCP1	0.97 – 3650.27
MCP2	0.94 – 231.72
MCP3	1.92 – 2081.28
MCP4	3.06 – 180.34
MDC	1.38 – 1257.87
MIF	25.82 – 24118.01
MIG	1.29 – 1422.29
MIP1alpha	0.35 – 415.70
MPIF1	1.28 – 1133.28
TNF Alpha	0.81 – 11573.72
